# Perspectives on Sexual Health Management among Tunisian Primary Care Physicians

**DOI:** 10.1192/j.eurpsy.2024.1601

**Published:** 2024-08-27

**Authors:** F. Baccar, B. Abassi, B. Amamou

**Affiliations:** ^1^Ibn Omrane, Razi Hospital, Manouba; ^2^Psychiatry, Fattouma Bourguiba, Monastir, Tunisia

## Abstract

**Introduction:**

Sexual health significantly influences individual well-being. It is thus crucial for primary care physicians to address these concerns effectively.

**Objectives:**

To evaluate the perspectives and approaches of primary care practitioners towards sexual health.

**Methods:**

A descriptive survey was disseminated to 350 primary care physicians via Google Forms in August 2022.

**Results:**

Of the respondents, 53.1% were female. The majority (71.4%) were affiliated with the public health sector, and over 75% were based in urban areas. All acknowledged the importance of addressing sexuality in their patients’ health. In this context, 62% spontaneously initiated discussions on the subject with their patients. Also, 72% noted that patients anticipate a regular dialogue about sexual health with their primary care provider. Over 90% believed in the value of addressing sexual dysfunctions more proactively, with 56% comfortable in leading such discussions. Additionally, 64% were inclined to include targeted questions on sexual health in their consultations. Notably, 77.6% expressed interest in creating specialized sexual health consultations in their practice. However, 54% felt unease in discussing sexual health with opposite-gender patients, and 82% lacked referrals to sexologists.

**Conclusions:**

Sexual health issues are not uncommon in general practice. Primary care providers play a vital role in counseling, screening, and educating patients on these concerns, necessitating specialized training to enhance patient management.

**Disclosure of Interest:**

None Declared

